# MiR-146a regulates regulatory T cells to suppress heart transplant rejection in mice

**DOI:** 10.1038/s41420-021-00534-9

**Published:** 2021-06-17

**Authors:** Jian Lu, Weiwei Wang, Peiyuan Li, Xiaodong Wang, Chao Gao, Baotong Zhang, Xuezhi Du, Yanhong Liu, Yong Yang, Feng Qi

**Affiliations:** 1 Department of General Surgery, Tianjin Medical University General Hospital, Anshan Road, Tianjin, 300052 China; 2 Department of General Surgery, Tianjin Medical University Baodi Clinical College, Guangchuan Road, Tianjin, 301800 China; 3 Department of Gastrointestinal Surgery, The First Affiliated Hospital, School of Medicine, Zhejiang University, Qingchun Road, Hangzhou, 310003 Zhejiang Province China

**Keywords:** Transplant immunology, Immunological disorders

## Abstract

Regulatory T cells (Tregs), which characteristically express forkhead box protein 3 (Foxp3), are essential for the induction of immune tolerance. Here, we investigated microRNA-146a (miR-146a), a miRNA that is widely expressed in Tregs and closely related to their homeostasis and function, with the aim of enhancing the function of Tregs by regulating miR-146a and then suppressing transplant rejection. The effect of the absence of miR-146a on Treg function in the presence or absence of rapamycin was detected in both a mouse heart transplantation model and cell co-cultures in vitro. The absence of miR-146a exerted a mild tissue-protective effect by transiently prolonging allograft survival and reducing the infiltration of CD4^+^ and CD8^+^ T cells into the allografts. Meanwhile, the absence of miR-146a increased Treg expansion but impaired the ability of Tregs to restrict T helper cell type 1 (Th1) responses. A miR-146a deficiency combined with interferon (IFN)-γ blockade repaired the impaired Treg function, further prolonged allograft survival, and alleviated rejection. Importantly, miR-146a regulated Tregs mainly through the IFN-γ/signal transducer and activator of transcription (STAT) 1 pathway, which is implicated in Treg function to inhibit Th1 responses. Our data suggest miR-146a controls a specific aspect of Treg function, and modulation of miR-146a may enhance Treg efficacy in alleviating heart transplant rejection in mice.

## Introduction

Organ transplantation is an effective treatment for patients with end-stage organ failure, but immune rejection after transplantation is still one of the main factors affecting the survival rate and quality of life of patients [1–3]. Although the application of various immunosuppressants may provide temporary symptom relief, unfortunately, these drugs have significant side effects [3–5].

Regulatory T cells (Tregs), which are characterized by the expression of the transcription factor forkhead box protein 3 (Foxp3), are a special population with immunoregulatory functions that inhibits the excessive activation of self-antigens, monitors the expansion of lymphocytes, and effectively suppresses excessive immune responses [6–8]. Adoptive transfer of Tregs effectively alleviates islet autoimmunity in mice [9]. Clinical trials have shown that the in vitro expansion and reinfusion of Tregs reduces the incidence of acute graft-versus-host disease and prolongs patient survival [10]. Therefore, increasing the number of Tregs or strengthening their immunoregulatory function may ameliorate rejection and induce immune tolerance. While a sufficient number of Tregs is induced in vitro at the early stage of clinical transplantation, several factors, such as low purity, insufficient function, and variable phenotype, limit the infusion of Tregs, and the adoptive transfer of Tregs alone is not sufficient to induce immune tolerance, suggesting that a different therapeutic approach is required to overcome these shortcomings [11–14].

MicroRNAs (miRNAs) are endogenous, noncoding, single-stranded RNA molecules with a length of approximately 22 nucleotides that are fully complementary to the 3’-UTR of their target mRNA, leading to mRNA cleavage or translational inhibition [15, 16]. MiRNAs play an important regulatory role in the homeostasis and function of Tregs in both normal and inflammatory environments [17–19]. Notably, miR-146a, which is expressed at high levels in Tregs relative to conventional CD4^+^ T cells [20], negatively regulates the activation of the NF-κB pathway by directly targeting TRAF6 and IRAK1 to control the inflammatory response [21, 22]. Other studies have shown that the expression and activation of signal transducer and activator of transcription (STAT) 1, one of the targets of miR-146a, is critical for Tregs to control interferon (IFN)-γ-mediated T helper cell type 1 (Th1) immune responses and associated inflammation [23]. However, the function and molecular mechanism of miR-146a expressed in Tregs in organ transplantation remain unclear.

This study found that the absence of miR-146a in Tregs promoted Treg survival but impaired their function. A miR-146a deficiency exerted a synergistic effect with IFN-γ neutralization on enhancing Treg function and alleviating rejection. Mechanistically, miR-146a regulated Tregs mainly through the IFN-γ/STAT1 pathway during rejection.

## Results

### Increased miR-146a expression in Tregs during rejection

**Fig. 1:**
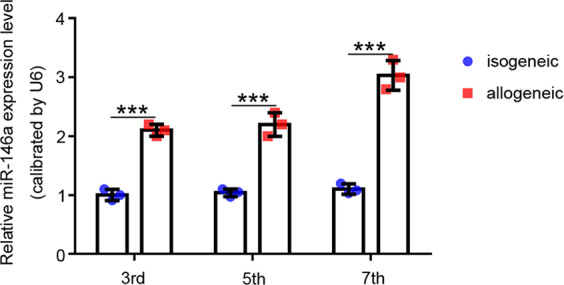
Differences in the expression levels of miR-146a in isogeneic and allogeneic transplants. The expression level of miR-146a in Tregs isolated from the spleens of isogeneic (WT → WT) and allogeneic transplant (BALB/c → WT) recipients on days 3, 5, and 7 after transplantation, *n* = 3. Data are presented as means ± SEM. ***p < 0.001.

We compared the expression of miR-146a in Tregs between the isogeneic transplant group (WT → WT) and the allogeneic transplant group (BALB/c→WT) to identify the regulatory effect of miR-146a on rejection. The results showed increased miR-146a expression in the Tregs of allogeneic transplant recipients on the 3rd, 5th, and 7th days after transplantation in a mouse heart transplantation model compared with those of the isogeneic transplant recipients (Fig. 1). Therefore, miR-146a is involved in the immune rejection process, and altering miR-146a expression may suppress rejection.

### The absence of miR-146a slightly inhibited rejection after transplantation

According to a previous report, miR-146a^−/−^ mice develop severe lymphatic and myelodysplastic syndromes at 6 months of age [23]; however, miR-146a CKO mice aged 6–8 weeks were used in our study, and no clinical signs of autoimmunity and inflammation were observed.

**Fig. 2:**
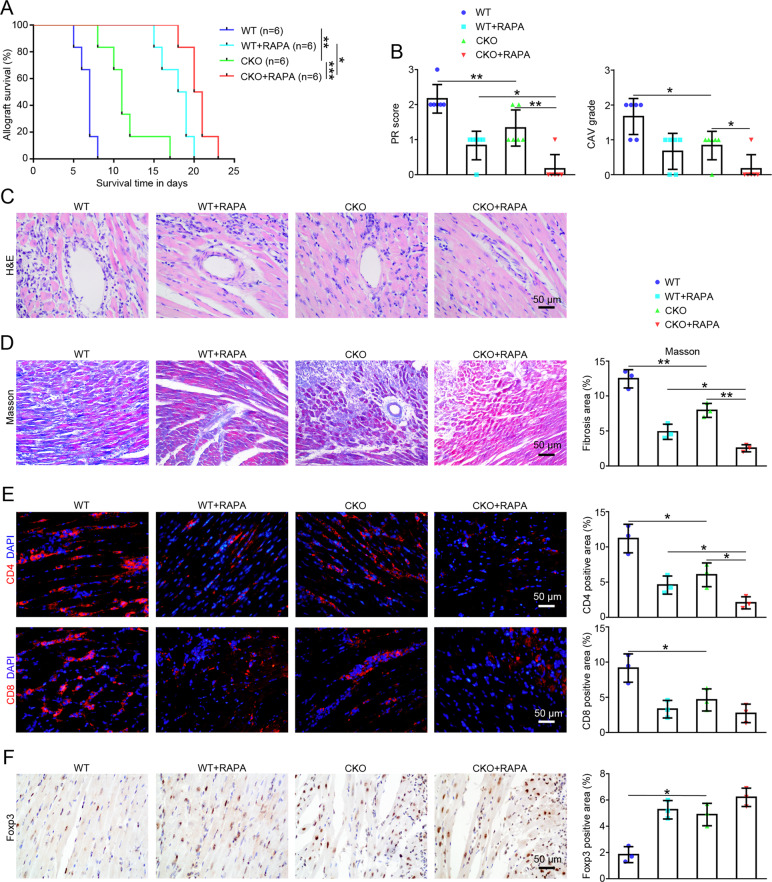
The effects of the absence of miR-146a on immune rejection after heart transplantation. **A** Kaplan-Meier analysis of the allograft survival time (log-rank test), *n* = 6. **B** Assessment of PR scoring and CAV grading of allografts using H&E staining according to the ISHLT guidelines, *n* = 6. **C** Histological analyses of the harvested allografts stained with H&E (×400; scale bars = 50 μm; *n* = 6) and **D** Masson’s trichrome (×200; scale bars = 50 μm; *n* = 3) were performed on day 5 after transplantation in the WT, WT+RAPA, CKO, and CKO+RAPA groups. **E** The infiltration of CD4^+^ and CD8^+^ T cells in the allografts of the WT, WT+RAPA, CKO, and CKO+RAPA groups on day 5 were detected using immunofluorescence staining (×400; scale bars = 50 μm), *n* = 3. **F** The infiltration of Tregs (Foxp3^+^) in the allografts of the WT, WT+RAPA, CKO, and CKO+RAPA groups on day 5 were detected using immunohistochemistry (×400; scale bars = 50 μm), *n* = 3. Data are presented as means ± SEM. *p < 0.05 and **p < 0.01. CKO indicates miR-146a conditional knockout mice. WT wild type, RAPA rapamycin.

After the generation of CKO mice, we first analyzed the survival time of the allografts to determine the effect of the absence of miR-146a on rejection in the mouse heart transplantation model. As shown in Fig. 2A, the median survival time (MST) in the CKO group was transiently prolonged from 7 days to 11 days (MST in the WT group, 7 days; MST in the CKO group, 11 days; *p* < 0.01), and the MST in the CKO+RAPA combination group was further prolonged to 20.5 days (MST in the CKO group, 11 days; MST in the CKO+RAPA group, 20.5 days; *p* < 0.001). The histological changes in the allografts on the 5th day after transplantation showed a low level of inflammatory cell infiltration, well-preserved myocardial structure, and mild vasculopathy in the CKO group compared with the WT group (Fig. 2C). Moreover, significant differences were observed in the PR scoring and CAV grading of H&E staining, and these changes were further alleviated in the CKO+RAPA group (Fig. 2B). In addition, Masson’s staining of the allografts on the 5th day after transplantation showed that the fibrotic area of allografts in the CKO group was reduced compared with the WT group, while the fibrotic area of allografts in the CKO+RAPA group was even further reduced compared with that of the CKO group (Fig. 2D). Based on these results, the absence of miR-146a transiently prolonged allograft survival time and reduced histological damage, and a synergistic effect was observed in combination with RAPA treatment.

The different degrees of infiltration by T lymphocytes and Tregs in the allografts are closely related to tissue damage. On the 5th day after transplantation, the proportions of infiltrated CD4^+^ and CD8^+^ T cells detected in the allografts using immunofluorescence staining were decreased in the CKO group (Fig. 2E), while the proportion of infiltrated Tregs (Foxp3^+^) detected using immunohistochemistry was increased (Fig. 2F) compared with the WT group. In addition, the combination of CKO+RAPA further reduced the proportion of CD4^+^ T cells compared with the CKO group (Fig. 2E).

**Fig. 3:**
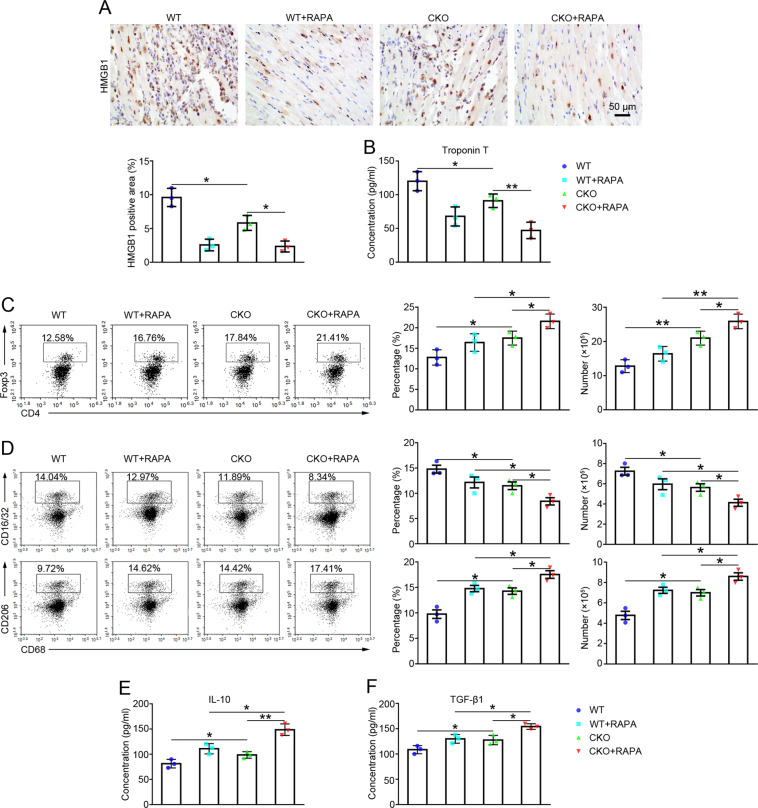
The effects of the absence of miR-146a on the function of allografts and immune response in recipients after heart transplantation. **A** The expression of HMGBI in the allografts was detected using immunohistochemistry (×400; scale bars = 50 μm) on day 5 in the WT, WT+RAPA, CKO, and CKO+RAPA groups, *n* = 3. **B** Serum levels of Troponin T were detected in recipient mice from all groups on day 5 using an ELISA. *n* = 3. **C** The flow cytometry analysis of the proportions and numbers of Tregs (CD4^+^ Foxp3^+^) in the spleens of recipients was performed on day 5 in all groups, *n* = 3. **D** The flow cytometry analysis of the proportions and numbers of M1 (CD16/32^+^ CD68^+^) and M2 (CD206^+^ CD68^+^) macrophages in the spleens of recipients was performed on day 5 in all groups, *n* = 3. **E**–**F** Serum levels of IL-10 and TGF-β1 were detected in recipients from all groups on day 5 using ELISAs, *n* = 3. Data are presented as means ± SEM. *p < 0.05 and **p < 0.01. CKO indicates miR-146a conditional knockout mice. WT wild type, RAPA rapamycin.

High mobility group box 1 (HMGB1) [24] and Troponin T [25] are important mediators of myocardial inflammation and heart failure. On the 5th day after transplantation, both HMGB1 expressions (Fig. 3A) detected in allografts using immunohistochemistry and serum Troponin T levels (Fig. 3B) detected using ELISAs were decreased in recipients in the CKO group compared with the WT group, and HMGB1 and Troponin T levels were both decreased in the CKO + RAPA group compared with the CKO group (Fig. 3A–B).

Thus, the absence of miR-146a slightly alleviates rejection, with synergistic therapeutic effects observed in combination with RAPA treatment.

### Effect of the absence of miR-146a on the immune response of transplant recipients

Flow cytometry was used to analyze each cell type in the recipient’s spleens on the 5th day after transplantation and to investigate the effect of miR-146a on the immune response in the recipients. As shown in Fig. 3C, the proportions and numbers of Tregs (CD4^+^Foxp3^+^) were increased in the CKO group compared with those in the WT group. The proportions and numbers of Tregs were further increased in the CKO+RAPA group compared with those in the CKO group.

Macrophage polarization was also assessed in the recipients. As shown in Fig. 3D, compared with those in the WT group, the proportions and numbers of M1 macrophages (CD16/32^+^CD68^+^) were decreased in the CKO group, while the proportions and numbers of M2 macrophages (CD206^+^CD68^+^) increased. The proportions and numbers of M1 macrophages in the CKO+RAPA group were further decreased, while the proportions and numbers of M2 macrophages were further increased compared with those in the CKO group.

In addition, the expression levels of inflammatory factors in the serum of recipient mice were detected using ELISAs. Compared with the WT group, the levels of the anti-inflammatory factors IL-10 and TGF-β1 were increased in the CKO group (Fig. 3E–F). CKO combined with RAPA further increased IL-10 and TGF-β1 levels compared with those in the CKO group (Fig. 3E–F).

These results suggest that the absence of miR-146a in Tregs not only increases the number of Tregs in transplant recipients but also promotes M2 macrophage polarization.

### The absence of miR-146a promoted the expansion of Tregs in vivo

**Fig. 4:**
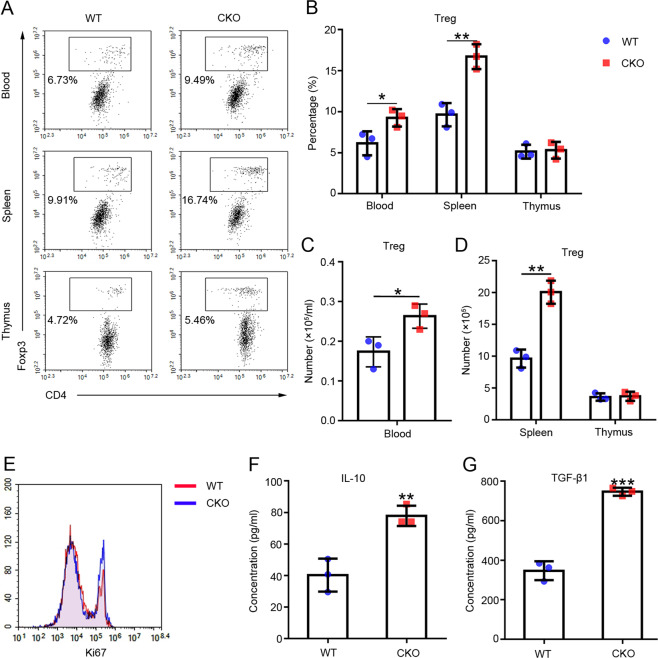
The effects of the absence of miR-146a on the proportion, levels of inflammatory factors, and Ki67 expression in Tregs from mice in different groups. Flow cytometry analysis of the **A**–**B** proportions and **C**–**D** numbers of Tregs (CD4^+^ Foxp3^+^) in the peripheral blood, spleen, and thymus of the WT or CKO mice, *n* = 3. **e** Flow cytometry analysis of the Ki67 expression level in Tregs isolated from the WT or CKO mouse spleens, *n* = 3. **F**–**G** Serum levels of IL-10 and TGF-β1 in WT or CKO mice were detected using ELISAs, *n* = 3. Data are presented as means ± SEM. *p < 0.05, **p < 0.01, and ***p < 0.001. CKO indicates miR-146a conditional knockout mice. WT wild type, Treg regulatory T cell.

We examined the proportions and numbers of Tregs in the peripheral blood, spleen, and thymus of the WT and CKO mice using flow cytometry to evaluate the effect of miR-146a on Treg proliferation. The results showed increased proportions (Fig. 4A–B) and numbers (Fig. 4C–D) of Tregs in the peripheral blood and spleen, but not in the thymus, of the CKO group compared with the WT group. Consistent with their increased proliferation, CKO Tregs expressed high levels of Ki67 (Fig. 4E).

Furthermore, we tested the expression levels of inflammatory cytokines in serum. The ELISA results showed increased levels of the anti-inflammatory cytokines IL-10 and TGF-β1 in the CKO group compared with their levels in the WT group (Fig. 4F–G).

These results are consistent with those obtained from transplant recipients, suggesting that the absence of miR-146a promotes Treg expansion in vivo.

### The absence of miR-146a in Tregs inhibited M1 macrophage polarization and promoted M2 macrophage polarization in vitro

**Fig. 5:**
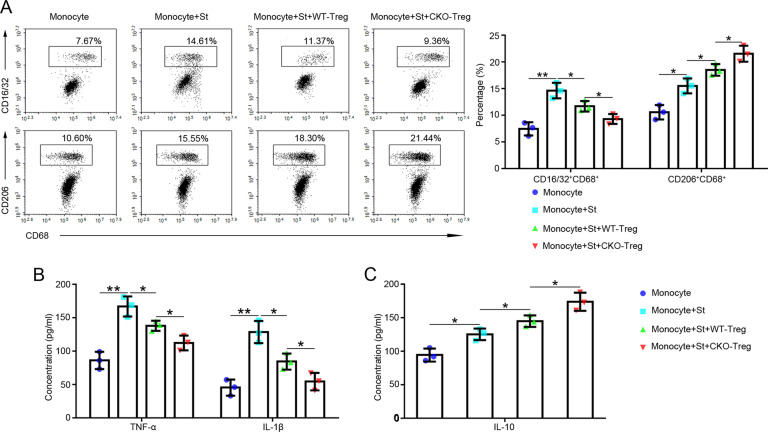
The effects of the absence of miR-146a in Tregs on macrophage polarization. **A** Monocytes from BALB/c spleens were co-cultured with or without WT-Tregs or CKO-Tregs and different stimuli for 72 h, and a flow cytometry analysis of the proportions of M1 (CD16/32^+^ CD68^+^) and M2 (CD206^+^ CD68^+^) macrophages was performed, *n* = 3. **B**–**C** The levels of TNF-α, IL-1β, and IL-10 in the supernatant of the co-cultures of monocytes and Tregs were detected using ELISAs. *n* = 3. Data are presented as means ± SEM. *p < 0.05 and **p < 0.01. St indicates the corresponding stimuli. CKO indicates miR-146a conditional knockout mice. WT-Treg and CKO-Treg indicate Tregs isolated from WT and miR-146a CKO mouse spleens, respectively. WT wild type, Treg regulatory T cell.

The effects of a miR-146a deficiency in Tregs on macrophage (M1, CD16/32^+^ CD68^+^ and M2, CD206^+^ CD68^+^) polarization were investigated in co-culture experiments in vitro. The proportion of each cell type was analyzed using flow cytometry. As shown in Fig. 5A, in the presence of stimuli, co-culturing monocytes with CKO Tregs significantly decreased the proportion of M1 macrophages and increased the proportion of M2 macrophages compared with co-culturing monocytes with WT Tregs. In addition, the levels of TNF-α and IL-1β secreted by M1 macrophages (Fig. 5B) and IL-10 secreted by M2 macrophages (Fig. 5C) were decreased and increased, respectively, in the CKO group compared with the WT group in the cell co-culture system. Based on these results, the absence of miR-146a enhanced the immunoregulatory effect of Tregs on macrophages.

### The absence of miR-146a in Tregs regulated CD4^+^ T cell behaviors in vitro

**Fig. 6:**
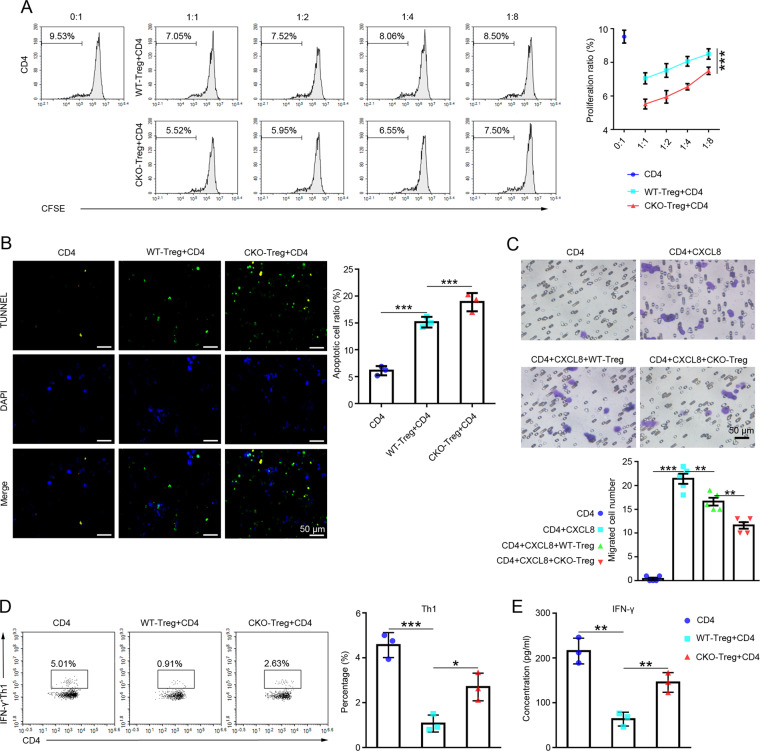
The effects of the absence of miR-146a in Tregs on CD4^+^ T cell behaviors. **A** Flow cytometry analysis of the proliferation rate of the CFSE-labeled CD4^+^ T cells cultured alone or co-cultured with WT-Tregs or CKO-Tregs for 72 h, *n* = 3. **B** Images of TUNEL staining (×400; scale bars = 50 μm) indicating the apoptosis of CD4^+^ T cells cultured alone or co-cultured with WT-Tregs or CKO-Tregs for 72 h, *n* = 3. **C** Images of the Transwell migration assay (×400; scale bars = 50 μm) of the CD4^+^T cells cultured alone or co-cultured with WT-Tregs or CKO-Tregs and CXCL8 for 72 h, *n* = 5. **D** Flow cytometry analysis of the differentiation of CD4^+^ T cell subsets (Th1) cultured alone or co-cultured with WT-Tregs or CKO-Tregs for 72 h, *n* = 3. **E** The level of IFN-γ in the co-culture supernatant was detected using an ELISA, *n* = 3. Data are presented as means ± SEM. *p < 0.05, **p < 0.01, and ***p < 0.001. CD4 indicates CD4^+^ T cells isolated from BALB/c spleens. CKO indicates miR-146a conditional knockout mice. WT-Treg and CKO-Treg indicate Tregs isolated from WT and miR-146a CKO mouse spleens, respectively. WT wild type, Treg regulatory T cell.

CD4^+^ T cells were co-cultured with Tregs isolated from the WT or CKO group to determine the effect of miR-146a on the immunosuppressive function of Tregs. The proliferation rate of CD4^+^ T cells was analyzed after co-culture with Tregs using flow cytometry. As shown in Fig. 6A, CKO Tregs significantly inhibited the proliferation of CD4^+^ T cells compared with WT Tregs.

The apoptosis of CD4^+^ T cells co-cultured with Tregs was detected using TUNEL staining. A higher apoptosis rate was observed for CD4^+^ T cells in the CKO group than in the WT group (Fig. 6B).

Moreover, the migration of CD4^+^ T cells co-cultured with Tregs was evaluated by performing Transwell experiments. CXCL8 was used to induce CD4^+^ T cell migration [26]. As shown in Fig. 6C, the CD4^+^ T cell migration efficiency was significantly decreased in the presence of CXCL8 in the CKO group compared with the WT group.

Further analysis of the differentiation of CD4^+^ T cells co-cultured with Tregs showed that the proportion of Th1 (CD4^+^IFN-γ^+^) cells (Fig. 6D) and the expression level of the Th1 signature cytokine IFN-γ (Fig. 6E) were elevated in the CKO group compared to the WT group.

These results suggest that the absence of miR-146a does not contribute to the overall suppressive function of Tregs, but rather appears to impair the ability of Tregs to control the Th1 responses.

### miR-146a regulated Tregs via the IFN-γ/STAT1 signaling pathway

**Fig. 7:**
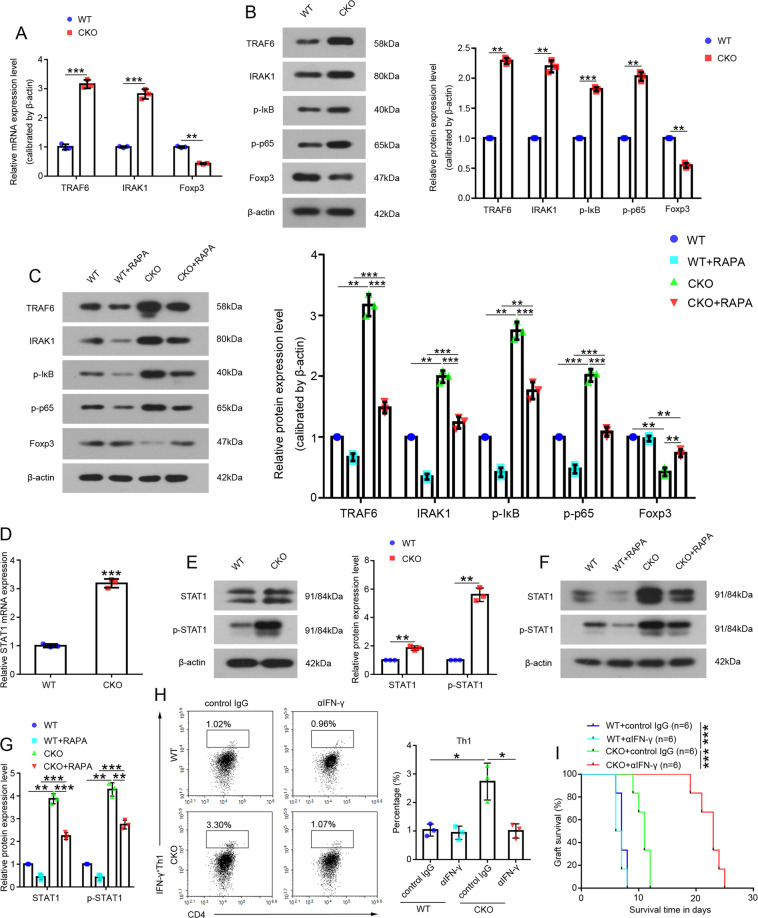
The related mechanism by which miR-146a regulates Tregs. **A** Expression of the TRAF6, IRAK1, and Foxp3 mRNAs in Tregs isolated from the WT mouse or CKO mouse spleens, *n* = 3. **B** Levels of the total TRAF6, IRAK1, and Foxp3 proteins and phosphorylated IκB and p65 proteins in Tregs isolated from the WT mouse or CKO mouse spleens, *n* = 3. **C** Levels of the total TRAF6, IRAK1, and Foxp3 proteins and phosphorylated IκB and p65 proteins in Tregs isolated from recipient’s spleens were detected on day 5 in the WT, WT+RAPA, CKO, and CKO+RAPA groups. *n* = 3. **D** The levels of the mRNA and **E** total and phosphorylated STAT1 protein in Tregs isolated from the WT or CKO mouse spleens. *n* = 3. **F**–**G** The total protein level and phosphorylation of STAT1 in Tregs isolated from the recipient’s spleens were analyzed on day 5 in the WT, WT+RAPA, CKO, and CKO+RAPA groups, *n* = 3. **H** CD4^+^ T cells from BALB/c spleens were co-cultured with Tregs isolated from the spleens of WT recipients or CKO recipients and treated with control IgG or αIFN-γ for 72 h, and then a flow cytometry analysis of the proportions of Th1 cells was performed. *n* = 3. **I** Kaplan-Meier analysis of the allograft survival time (log-rank test). *n* = 6. Data are presented as means ± SEM. *p < 0.05, **p < 0.01, and ***p < 0.001. CKO indicates miR-146a conditional knockout mice. WT wild type, RAPA rapamycin.

We measured NF-κB expression in Tregs to elucidate the mechanism by which miR-146a regulates Tregs. Levels of the TRAF6 and IRAK1 mRNAs and proteins and levels of phosphorylated IκB and p65, markers of NF-κB activation, were increased in CKO Tregs (Fig. 7A–B). In addition, levels of the TRAF6 and IRAK1 proteins and phosphorylated IκB and p65, markers of NF-κB activation, were all increased in Tregs from the CKO recipient group compared to those from the WT recipient group in animal models (Fig. 7C). Foxp3 is a lineage-specific transcription factor responsible for the differentiation and function of Tregs [27, 28]. However, Foxp3 was downregulated in CKO Tregs (Fig. 7A–B) and CKO recipient Tregs (Fig. 7C) compared with WT Tregs and WT recipient Tregs, respectively, suggesting that expression and activation of NF-κB were not responsible for Treg function.

Because a single miRNA regulates multiple targets simultaneously, we next explored the molecular mechanisms by which miR-146a-deficient Tregs mediate Th1 responses, and found that STAT1 in the IFN-γ/STAT1 pathway was expressed at high levels and activated in CKO Tregs (Fig. 7D–E) and CKO recipient Tregs (Fig. 7F–G) compared with WT Tregs and WT recipient Tregs, respectively.

However, when IFN-γ/STAT1 function was inhibited by αIFN-γ, the CKO Tregs+αIFN-γ significantly reduced the proportion of Th1 cells compared to CKO Tregs+control IgG in vitro (Fig. 7H), and miR-146a deficiency combined with IFN-γ neutralization significantly prolonged allograft MST from 11 days to 23 days in vivo (MST in the CKO+ control IgG group, 11 days; MST in the CKO+αIFN-γ group, 23 days; *p* < 0.001; Fig. 7I).

## Discussion

Immune rejection after organ transplantation is a difficult problem in immunology [29]. As our understanding of immune rejection has improved, emerging therapeutic strategies (including blocking costimulatory signals and inducing donor antigen tolerance) have entered the clinical arena [30–32]. The use of Tregs is one of the most promising natural mechanisms and strategies for inducing tolerance [33]. Given the important regulatory role of miRNAs in innate and adaptive immune cell development and function [34], our study suggested a new strategy based on miRNAs to regulate Tregs. The observed results are described below. First, the absence of miR-146a slightly inhibited rejection in animal models, and the therapeutic effect was more significant in the presence of RAPA treatment. Second, the absence of miR-146a promoted Treg expansion but impaired their function. Third, the absence of miR-146a in combination with IFN-γ treatment reversed the impaired function of Tregs and further alleviated rejection. Finally, miR-146a regulated Tregs through the IFN-γ/STAT1 pathway.

Abnormal inhibition or activation of T lymphocytes induces an abnormal immune response; however, the recruitment and activation of T lymphocytes, the participation of Tregs, and their inflammatory factors are involved in immune rejection [35, 36]. The balance between these two cell types is very important for inducing immune tolerance and moderately controlling immune rejection. Similarly, we observed changes in the Treg number and levels of anti-inflammatory factors (IL-10 and TGF-β1) in response to immune signaling in the absence of miR-146a during rejection.

Macrophages are the first line of defense in tissue immune responses [37]. Differently activated macrophages (M1 and M2) exhibit different phenotypes and functions. M1 macrophages tend to secrete proinflammatory cytokines and aggravate tissue damage, while M2 macrophages counteract the M1 response by reducing inflammation and repairing damaged tissue [38]. Tian et al. indicated that Tregs are important inhibitors of macrophage activation [39], and subsequent studies found that Tregs induce macrophage activation in an alternative manner, as reflected by inhibiting M1 polarization while promoting M2 polarization [40–42]. In this study, the absence of miR-146a enhanced the ability of Tregs to promote macrophage polarization to the M2 type in vivo and in vitro and inhibited M1 macrophage polarization. Increased M2 macrophage polarization promotes the inhibition of rejection [43]. Therefore, we propose that the extent of macrophage activation is closely related to the outcome of the allografts.

The absence of miR-146a in Tregs exerted a mild suppressive effect, prolonging the allograft MST from only 7 days to 11 days in animal models. We investigated the regulatory effect of miR-146a-deficient Tregs on CD4^+^ T cells in vitro to explain this phenomenon. Subsequently, in the co-cultures of Tregs and CD4^+^ T cells in vitro, the absence of miR-146a enhanced Treg-mediated inhibition of CD4^+^ T cell proliferation and migration and promotion of CD4^+^ T cell apoptosis but impaired the ability of Tregs to suppress Th1 differentiation, suggesting that the absence of miR-146a is detrimental to a specific aspect of Treg suppressor function, consistent with a publication by Lu et al. [23]. Furthermore, these authors observed that miR-146a^−/−^ mice develop a pathological Th1-responsive disease, namely, a lethal breakdown of immune tolerance due to the production of large amounts of the Th1 signature cytokine IFN-γ by miR-146a-deficient T cells and the failure of miR-146a-deficient Tregs to control IFN-γ responses [23]. However, in our study, the absence of miR-146a specifically in Tregs but not in T cells avoided the effect of miR-146a on T cells in miR-146a CKO mice; therefore, further investigations are needed to determine whether CKO mice exhibit an IFN-γ-mediated Th1 pathological disease.

Foxp3 is a key regulator of Tregs that maintains Treg function and lineage stability by controlling the expression of various gene regulators, including miRNAs [44, 45]. According to previous studies, NF-κB positively influences Treg function, at least in part, by regulating the transcription of Foxp3 [46, 47]. Liu et al. showed that Foxp3 inhibits tumor cell proliferation and promotes apoptosis by regulating the miR-146a/NF-κB axis in breast cancer and prostate cancer [48, 49]. However, researchers have not clearly determined whether miR-146a and NF-κB are involved in regulating Tregs during rejection. In this study, NF-κB expression and activation were increased in miR-146a-deficient Tregs, while Foxp3 expression was decreased, suggesting that miR-146a does not function through NF-κB to modulate Foxp3 expression and Treg function. In addition, the changes in Treg function in response to miR-146a deficiency may not only result from NF-κB but also because of the increased expression of other target genes of miR-146a, such as STAT1 [23]. In our study, we also observed increased expression and activation of the IFN-γ/STAT1 in miR-146a-deficient Tregs, which resulted in a failure of Tregs to control the Th1 responses. When IFN-γ/STAT1 function was inhibited, miR-146a-deficient Tregs restricted Th1 responses and prolonged allograft survival in the presence of αIFN-γ. Therefore, miR-146a-mediated regulation of Tregs was mainly dependent on the IFN-γ/STAT1 rather than NF-κB during rejection. The dominance of the increased expression and activation of the IFN-γ/STAT1 in miR-146a-deficient Tregs may have masked the effect of NF-κB.

Interestingly, the miR-146a deficiency increased the Treg number but impaired their functions. When the impaired function of miR-146a-deficient Tregs was reversed by αIFN-γ, the increase in Treg number was not affected by IFN-γ blockade [23], showing that changes in the function and number of Tregs were not uniform in the absence of miR-146a. On the other hand, in autoimmune diseases such as rheumatoid arthritis, a high frequency of Tregs is enriched in inflammation sites, but inflammatory conditions force the Treg function and phenotype to become unstable, which may accelerate autoimmune inflammation [50]. These experiments imply that Treg metabolism and suppressor functions are regulated by different molecular mechanisms.

## Conclusions

In conclusion, miR-146a regulates Tregs mainly through the IFN-γ/STAT1 pathway during rejection in mouse heart transplantation recipients, which is closely related to the ability of Tregs to suppress Th1 responses. The absence of miR-146a in Tregs combined with IFN-γ neutralization significantly inhibited Th1 responses, prolonged allograft survival, and alleviated rejection. This study showed the Treg-mediated post-transplant immune tolerance from the perspective of regulating a single miRNA, miR-146a, providing new insights into Treg function and a new strategy for improving the function of Tregs to suppress clinical rejection.

## Materials and methods

### Animals

Wild-type C57BL/6 (WT) and BALB/c mice weighing 20–25 g and aged 6 to 8 weeks were purchased from the Experimental Animal Center of the Chinese Academy of Medical Sciences (Beijing, China). The miR-146a^flox/flox^ (Stock No.: 021475) and Foxp3^YFP-Cre^ (Stock No.: 016959) mice on a C57BL/6 background were purchased from The Jackson Laboratory (Bar Harbor, ME, USA). For the generation of miR-146a-conditional knockout mice (CKO; Fig. S1), miR-146a^flox/flox^ and Foxp3^YFP-Cre^ mice were interbred as described in a previous report [51]. All animals were matched for sex and age, randomly assigned to each group, and included in the analysis. All the experimental procedures followed protocols approved by the Animal Care and Use Committee of Tianjin Medical University General Hospital (Ethical Approval No.: IRB2015-YX-009).

### Mouse heart transplantation model

Intraabdominal heterotopic cardiac transplantation was performed as previously described [52]. Briefly, donor hearts from WT or BALB/c mice were transplanted into WT or CKO mouse recipients by microsurgery. In the isogeneic transplant group, both the donor and recipient were WT mice. The donor in the allogeneic group was a BALB/c mouse and the recipient was WT or CKO mouse. Then, all allogeneic transplant recipients were randomly divided into 4 groups (*n* = 6) as follows: BALB/c → WT, BALB/c → WT + rapamycin (RAPA), BALB/c → CKO, and BALB/c → CKO+RAPA. Some recipient groups were subcutaneously injected with RAPA at a dose of 2 mg daily for 13 days. Allograft survival was assessed daily by palpation. The sera, spleens, and allografts of the recipients were collected on the 5th day after transplantation. Tregs from the recipient’s spleens were sorted using the CD4^+^ CD25^+^ Regulatory T Cell Isolation Kit (Miltenyi, Bergisch Gladbach, Germany) on the 3rd, 5th, and 7th days after transplantation.

### Evaluation of heart allograft rejection

Allografts from the recipients of each group were collected, fixed with 4% formalin, dehydrated, embedded in paraffin, and cut into 4-μm sections. Histological changes were observed using hematoxylin-eosin (H&E; Solarbio, Beijing, China) staining, and the myocardial fibrosis area was observed using Masson’s trichrome staining (Solarbio). Based on H&E staining, parenchymal rejection (PR) [53] and cardiac allograft vasculopathy (CAV) [54] of the allografts were graded according to the guidelines recommended by the International Society for Heart and Lung Transplantation (ISHLT).

For immunofluorescence staining, the sections were hydrated and blocked with normal goat serum. After incubation with the primary antibodies against CD4 (1:100; Catalog No.: ab183685; Abcam, UK) and CD8 (1:100; Catalog No.: 14-0081-82; eBioscience, USA) at 4 °C overnight, the sections were incubated with a fluorescent secondary antibody at room temperature and observed under a fluorescence microscope. The sections were evaluated by two pathologists in a double-blind manner.

For immunohistochemistry, sections were dewaxed, hydrated, subjected to antigen retrieval, and blocked with normal goat serum at room temperature for 15 min. After incubation with primary antibodies against Foxp3 (1:200; Catalog No.: ab215206; Abcam, UK) and HMGB1 (1:200; Catalog No.: 6893; CST, USA) at 4 °C overnight, the sections were incubated with a biotinylated secondary antibody and stained with DAB substrate. After counterstaining with hematoxylin, all sections were observed under an optical microscope.

The serum level of Troponin T in recipients was determined using enzyme-linked immunosorbent assay (ELISA) kits (Reddot Biotech, Canada) according to the manufacturer’s instructions.

### Evaluation of the immune response in recipient mice

The spleens were collected from the recipients in each group, ground, and filtered through a 40-mesh screen. Red blood cells were lysed with red blood cell lysis buffer (Solarbio), and then the remaining cells were washed twice and resuspended in phosphate-buffered saline (PBS; Solarbio). Fluorescent dye-conjugated antibodies were added to 10^5^ cells in 100 µl of PBS and incubated in the dark for 30 min. The following antibodies were used for the flow cytometry analysis: anti-CD4-FITC (Catalog No.: 100405; BioLegend, USA), anti-Foxp3-PE (Catalog No.: 126403; BioLegend, USA), anti-CD16/32-FITC (Catalog No.: 101305; BioLegend, USA), anti-CD206-FITC (Catalog No.: 141703; BioLegend, USA), and anti-CD68-APC (Catalog No.: 137007; BioLegend, USA) antibodies.

Serum levels of interleukin (IL)−10 and transforming growth factor (TGF)-β1 in recipients were determined using ELISA kits (DAKEWE, Beijing, China) according to the manufacturer’s instructions.

### Assessment of the immune microenvironment

The spleen, peripheral blood, and thymus of WT and CKO mice were collected, and cell suspensions were prepared. Next, the cells were labeled with anti-CD4-FITC (Catalog No.: 100405; BioLegend, USA) and anti-Foxp3-PE (Catalog No.: 126403; BioLegend, USA) antibodies. The percentage of positive cells was measured using flow cytometry.

Serum levels of IL-10 and TGF-β1 were determined using ELISA kits (DAKEWE) according to the manufacturer’s instructions.

### Isolation of Tregs and evaluation of Ki67 expression

Tregs from WT and CKO mouse spleens were sorted using a CD4^+^ CD25^+^ Regulatory T Cell Isolation Kit (Miltenyi) and labeled with the AlexaFluor®488 anti-mouse/human Ki67 antibody (Catalog No.: 151204; BioLegend, USA). The expression level of Ki67 in Tregs was measured using flow cytometry.

### Evaluation of macrophage polarization in vitro

Monocytes from BALB/c spleen samples were sorted using a Mouse Monocyte Isolation Kit (Miltenyi), co-cultured with Tregs at monocyte:Treg ratios of 2:0 and 2:1, and exposed to different stimuli to determine the effect of a miR-146a deficiency in Tregs on macrophage polarization. Macrophage type 1 (M1) cells were stimulated with lipopolysaccharide (LPS; 10 µg/ml; Solarbio) + interferon (IFN)-γ (20 ng/ml; PeproTech), and macrophage type 2 (M2) cells were stimulated with LPS (10 µg/ml; Solarbio)+IL-4 (50 ng/ml; PeproTech). After 72 h of co-culture, the cells from each group were harvested and labeled with anti-CD16/32-FITC (Catalog No.: 101305; BioLegend, USA), anti-CD206-FITC (Catalog No.: 141703; BioLegend, USA), and anti-CD68-APC (Catalog No.: 137007; BioLegend, USA) antibodies, and the ratios of various types of cells were detected using flow cytometry.

The levels of tumor necrosis factor (TNF)-α, IL-1β, and IL-10 in the supernatant of co-cultured monocytes and Tregs were determined using ELISA kits (DAKEWE) according to the manufacturer’s instructions.

### Isolation of CD4^+^ T cells and stimulation with CD3/CD28 microbeads

CD4^+^ T cells from BALB/c spleens were sorted with the mouse CD4 (L3T4) MicroBeads Isolation Kit (Miltenyi). The purified CD4^+^ T cells were labeled with carboxyfluorescein succinimidyl amino ester (CFSE) (100 μg/ml; BD Biosciences, CA, USA). CD4^+^ T cells were stimulated with CD3/CD28 microbeads (bead-to-cell ratio of 1:1; Miltenyi) for 72 h.

### Evaluation of CD4^+^ T cell behaviors in vitro

We performed lymphocyte co-culture experiments to examine the effect of the absence of miR-146a in Tregs on CD4^+^ T cell behaviors. For the CD4^+^ T cell proliferation assay, Tregs and CFSE-labeled CD4^+^ T cells were co-cultured for 72 h at Treg: CD4^+^ T cell ratios of 0:1, 1:1, 1:2, 1:4, and 1:8, and the proliferation response of CD4^+^ T cells was assessed using flow cytometry.

For the CD4^+^ T cell apoptosis assay, Tregs and CD4^+^ T cells were seeded at Treg:CD4^+^ T cell ratios of 0:1 and 1:1 into the upper and lower chambers of 0.4-μm polycarbonate membrane Transwell inserts (Corning, NY, USA), respectively. After 72 h of co-culture, CD4^+^ T cells were harvested and cultured on 13-mm-diameter glass coverslips in 24-well plates at 37 °C for 15 min, and then the cells were permeabilized with 0.1% Triton X-100 (Beyotime, Shanghai, China) for 15 min at room temperature. After these basic procedures, the cells were subjected to TUNEL (Roche, Basel, Switzerland) staining according to the manufacturer’s instructions, and images were obtained with a confocal microscope (Olympus, Tokyo, Japan).

For the CD4^+^ T cell migration assay, after the Tregs and CD4^+^ T cells were co-cultured at Treg:CD4^+^ T cell ratios of 0:1 and 1:1 in a 0.4-μm Transwell culture system for 72 h as described above, CD4^+^ T cells translocated to the upper chambers of 8-μm polycarbonate membrane Transwell inserts (Corning). RPMI-1640 medium containing CXCL8 (150 ng/ml; PeproTech) was added to the lower chamber. After culturing for 4 h, the medium was discarded, and the cells were stained with crystal violet (Amresco, Solon, OH, USA) and imaged with a microscope (Olympus). The number of migrated cells was determined in the images.

For the CD4^+^ T cell differentiation assay, Tregs and CD4^+^ T cells were co-cultured for 72 h at Treg:CD4^+^ T cell ratios of 0:1 and 1:1. The cells were harvested and labeled with anti-CD4-APC (Catalog No.: 100411; BioLegend, USA), anti-IFN-γ-PE (Catalog No.: 505807; BioLegend, USA) antibodies, and the ratios of CD4^+^IFN-γ^+^ Th1 cells were detected using flow cytometry.

According to the manufacturer’s instructions, the expression level of IFN-γ in the co-culture supernatant was detected using ELISA kits (DAKEWE).

### Reverse transcription-polymerase chain reaction (RT-PCR)

Total RNA was extracted with the TRIpure kit (BioTeke, Beijing, China) and the concentration of total RNA was measured using a NANO 2000 spectrophotometer (Thermo Fisher Scientific, Waltham, MA, USA). The cDNA templates were synthesized using the Super M-MLV Reverse Transcriptase Kit (BioTeke) and amplified with the SYBR Green method in an Exicycler™ 96 Fluorescent Quantitative Analyzer (Bioneer, Daejeon, Korea). The primer sequences were as follows: miR-146a, forward: 5-TGAGAACTGAATTCCATGGGTT-3 and reverse: 5-GCAGGGTCCGAGGTATTC-3; Foxp3, forward: 5-AGTGCTTTGTGCGAGTGG-3 and reverse: 5-AAGGGCAGGGATTGGAG-3; TRAF6, forward: 5-GCAGAGGAATCACTTGGCACGAC-3 and reverse: 5-ATCGCACGGACGCAAAGCA-3; IRAK1, forward: 5-ACAGAGGTGGAACAGCTATCA-3 and reverse: 5-TGGGCAAGAAGCCATAAAC-3; STAT1, forward: 5-CACGCCTTTGGGAAGTATTA-3 and reverse: 5-GAAGCAGGTTGTCTGTGGTCT-3; U6, forward: 5-CGCAAGGATGACACGCAAAT-3 and reverse: 5-GCAGGGTCCGAGGTATTC-3; and β-actin, forward: 5-CTGTGCCCATCTACGAGGGCTAT-3 and reverse: 5-TTTGATGTCACGCACGATTTCC-3.

### Western blot assay

Total protein was obtained using RIPA lysis buffer, and approximately 30 µg of total protein in each sample were separated on SDS/PAGE gels in an electrophoresis system (Bio-Rad, Mini-Protein Tetra System) and transferred to a PVDF membrane (Millipore, Billerica, MA, USA). The membrane was blocked with a TBST (TBS/0.15% Tween 20) solution containing 5% skim milk powder at room temperature for 1 h, the solution was discarded, and the PVDF membrane was washed three times with the TBST solution, followed by overnight probing at 4 °C with anti-Foxp3 (1:1000; Catalog No.: ab215206; Abcam, UK), anti-TRAF6 (1:5000; Catalog No.: ab33915; Abcam, UK), anti-IRAK1 (1:1000; Catalog No.: ab238; Abcam, UK), anti-p-IκB (1:1000; Catalog No.: 2859T; CST, USA), anti-p-p65 (1:1000; Catalog No.: 3033T; CST, USA), anti-STAT1 (1:1000; Catalog No.: 14994S; CST, USA), anti-p-STAT1 (1:1000; Catalog No.: 7649S; CST, USA), and anti-β-actin (1:5000; Catalog No.: 4970T; CST, USA) antibodies. The membrane was rinsed, incubated with diluted horseradish peroxidase (HRP)-labeled secondary antibody (1:50000; Catalog No.: 7071T; CST, USA) for 45 min at room temperature, and immunoreactive protein bands were detected using an enhanced chemiluminescence assay kit (Millipore). Images were acquired with a G-Box system (Syngene, Frederick, MD, USA) and analyzed with ImageJ software.

### Evaluation of IFN-γ neutralization

For this experiment, 0.5 mg of IFN-γ neutralizing antibody (αIFN-γ; Catalog No.: BE0055; BioXCell, USA) or isotype-matched control IgG was injected into WT recipient or CKO recipient mice via the tail vein 1 day before and 1 and 4 days after transplantation. Tregs from the recipient’s spleens were sorted using a CD4 + CD25 + Regulatory T Cell Isolation Kit (Miltenyi) on the 5th day after transplantation and co-cultured with CD4^+^T cells from BALB/c spleens at Treg:CD4 ratio of 1:1. After 72 h of co-culture, the cells were harvested and labeled with anti-CD4-APC (Catalog No.: 100411; BioLegend, USA) and anti-IFN-γ-PE (Catalog No.: 505807; BioLegend, USA) antibodies, and the ratios of Th1 cells were detected using flow cytometry. Allograft survival was assessed daily by palpation.

### Flow cytometry

Cell surface and intracellular staining assays were performed as previously described [55]. Cell fluorescence was detected using a NovoCyte Flow cytometer (ACEA Bioscience, San Diego, CA, USA), and data were analyzed using NovoExpress software (ACEA Bioscience).

### Statistical analysis

The investigator and the outcome assessment were blinded during the experiment. SPSS 22.0 (IBM SPSS Statistics, Armonk, NY, USA) was used for statistical analyses. All data are presented as the means ± standard errors of the means (mean ± SEM), and comparisons between groups were performed using a two-sided Student’s *t*-test. The differences among the groups were analyzed using one-way ANOVA. Kaplan-Meier survival curves were used to analyze allograft survival. *P* values < 0.05 were considered statistically significant. All experiments were performed three times, and the sample size of mice was *n* = 3–6 per group.

## Supplementary information

Supplementary Figure Legends

Fig. S1
