# Supplementary material for: MiR-146a regulates regulatory T cells to suppress heart transplant rejection in mice
Source: Cell Death Discov. 2021 Jun 17;7:165. doi: 10.1038/s41420-021-00534-9 (PMC8257678; doi:10.1038/s41420-021-00534-9)
Supplement: Supplementary file 1 — Supplementary Figure Legends [file 41420_2021_534_MOESM1_ESM.docx]

**Supplementary Figure Legends**

**Fig. S1 Identification of miR-146a CKO mice. a, b** The miR-146a flox and Foxp3 cre genotypes were identified by PCR in this study. **c** Primer sequences for PCR identification of miR-146a flox and Foxp3 cre genotypes. **d** miR-146a was low expressed in Tregs but not in Tconvs in peripheral blood, spleen, and thymus of CKO mice. Data are represented as mean±SEM. ****p*<0.001. flox/+ and flox/flox indicate the heterozygous allele and the pure allele of miR-146a flox, respectively. H_2_O indicates water as a negative control. CKO indicates miR-146a conditional knockout mice. Abbreviations: WT, wild type; miR-146a, microRNA-146a. Treg, regulatory T cell; Tconv, conventional T cell.
