# Supplementary figures and images for: MiR-146a regulates regulatory T cells to suppress heart transplant rejection in mice
Source: Cell Death Discov. 2021 Jun 17;7:165. doi: 10.1038/s41420-021-00534-9 (PMC8257678; doi:10.1038/s41420-021-00534-9)

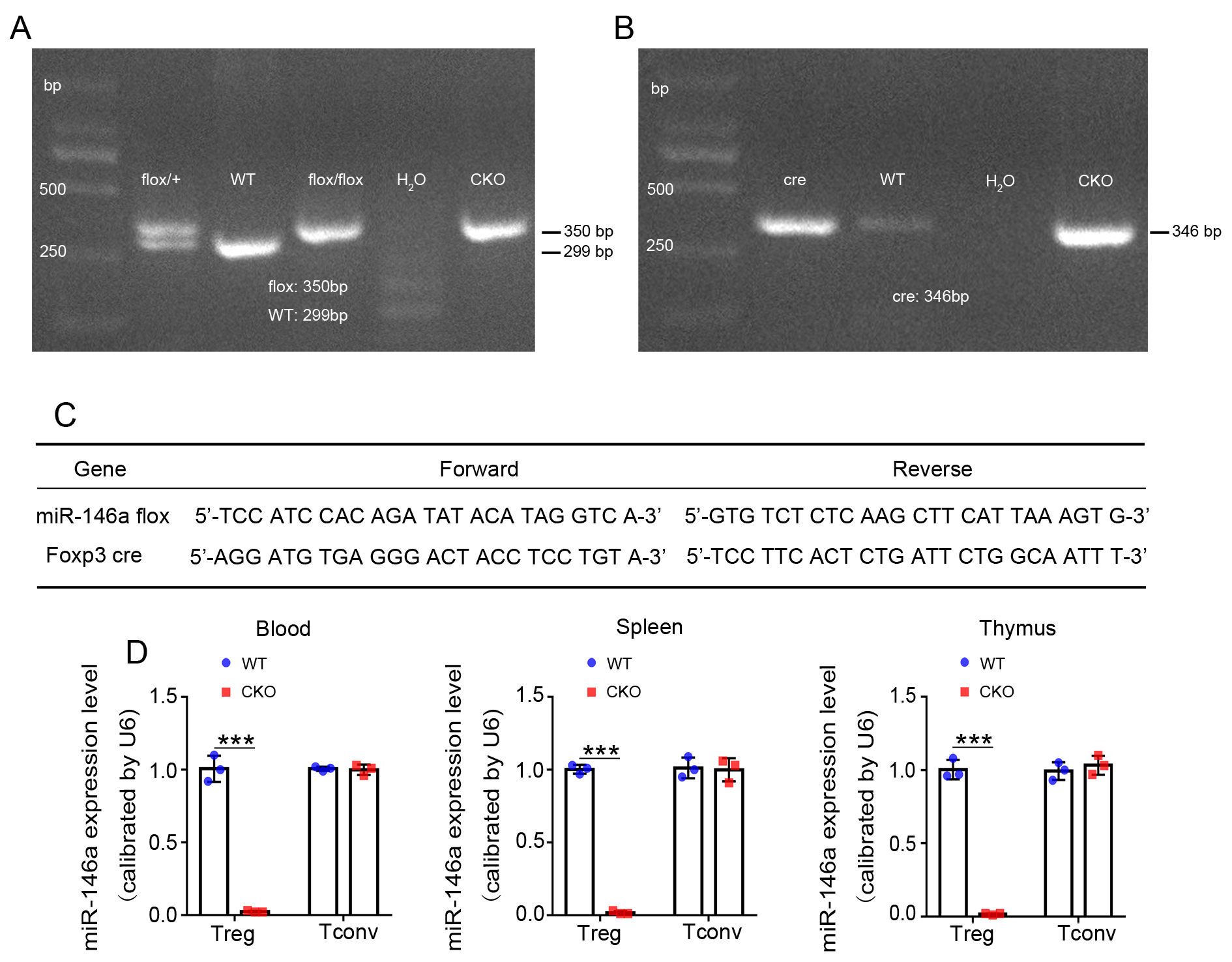

Supplement: Supplementary file 2 — Fig. S1 [file 41420_2021_534_MOESM2_ESM.tif]
